# Glycemic Index, Glycemic Load and Mammographic Breast Density: The EPIC Florence Longitudinal Study

**DOI:** 10.1371/journal.pone.0070943

**Published:** 2013-08-07

**Authors:** Giovanna Masala, Melania Assedi, Benedetta Bendinelli, Ilaria Ermini, Daniela Occhini, Sabina Sieri, Furio Brighenti, Marco Rosselli del Turco, Daniela Ambrogetti, Domenico Palli

**Affiliations:** 1 Molecular and Nutritional Epidemiology Unit, Cancer Research and Prevention Institute (ISPO), Florence, Italy; 2 Epidemiology and Prevention Unit, Department of Preventive & Predictive Medicine, Fondazione IRCCS Istituto Nazionale dei Tumori, Milan, Italy; 3 Department of Public Health, Università di Parma, Parma, Italy; 4 European School of Oncology ESO (Consultant), Milan, Italy; 5 Cancer Screening Unit, Cancer Research and Prevention Institute (ISPO), Florence, Italy; Health Canada and University of Ottawa, Canada

## Abstract

A few studies have evaluated the association between diet and mammographic breast density (MBD) and results are inconsistent. MBD, a well-recognized risk factor for breast cancer, has been proposed as a marker of cumulative exposure to hormones and growth factors. Diets with a high glycemic index (GI) or glycemic load (GL) may increase breast cancer risk, via an effect on the insulin-like growth factor axis. We have investigated the association between carbohydrate intake, GI, GL and MBD in a prospective study. We identified a large series of women, in the frame of the EPIC-Florence cohort, with a mammogram taken five years after enrolment, when detailed information on dietary and lifestyle habits and anthropometric measurements had been collected. Mammograms have been retrieved (1,668, 83%) and MBD assessed according to Wolfe’s classification. We compared women with high MBD (P2+DY Wolfe’s categories) with those with low MBD (N1+P1) through logistic models adjusted for age, education, body mass index, menopause, number of children, breast feeding, physical activity, non-alcohol energy, fibers, saturated fat and alcohol. A direct association between GL and high MBD emerged in the highest quintile of intake in comparison with the lowest quintile (OR = 1.73, 95%CI 1.13–2.67, p for trend = 0.048) while no association with glycemic index was evident. These results were confirmed after exclusion of women reporting to be on a diet or affected with diabetes, and when Hormone Replacement Therapy at the date of mammographic examination used to assess MBD was considered. The effect was particularly evident among leaner women, although no interaction was found. A positive association was suggested for increasing simple sugar and total carbohydrates intakes limited to the highest quintiles. In this Italian population we observed an association between glycemic load, total and rapidly absorbed carbohydrates and high MBD. These novel results warrant further investigations.

## Introduction

Mammographic breast density (MBD) is a well-recognized risk factor for breast cancer (BC). The positive association between MBD and BC risk, first proposed by Wolfe [1], has been consistently reported by studies using either a qualitative assessment of breast density or a quantitative assessment of the area of the breast showing radiographic density [2], [3].

It has been proposed that MBD may be considered a marker of cumulative exposure to hormones and other growth factors [4] that in turn might affect BC risk. Some studies have shown an association between circulating levels of insulin-like growth factor I (IGF-I) and increased levels of mammographic density [5].

High circulating glucose, insulin resistance and obesity appear to be associated with increased risk of BC. Diets high in carbohydrates may result in chronically elevated insulin concentrations and may affect BC risk by stimulation of insulin receptors or IGF-I mediated mitogenesis [6], [7]. Glycemic index (GI) provides a physiological classification of carbohydrate containing foods according to their effect on post-prandial blood glucose concentrations in comparison to a standard (glucose or bread) [8], [9]. Glycemic load (GL), the product of the specific GI and the total carbohydrate content of each food, is a global indicator of the glycemic response and insulin demand induced by a serving of that food. The two may be calculated as average values based on reported foods consumed in the usual diet in order to describe the dietary profile of a specific subject.

Several studies have investigated GI and GL as potential risk factors for BC with contrasting results. In a review and meta-analysis including 6 prospective cohort studies no clear evidence of an association between GI and GL and BC was observed [10]. In a recent meta-analysis including four additional prospective studies, high GI was associated to an increased risk of BC in both pre- and post-menopausal women [11], while no association was apparent for GL. However a study carried out recently in the Italian section of the European Prospective Investigation into Cancer and Nutrition (EPIC) study has reported a positive association between increased GL and BC risk [12].

Only a few studies have investigated the effects of dietary carbohydrates on MBD, mostly with inconsistent results [13]–[18] but, so far, the specific associations with GL and GI have not been investigated. We have previously carried out analyses on dietary determinants of high MBD in a prospective study, with over 1,600 Italian women, and no specific effect of high carbohydrate intake, overall and separately for simple sugars and starches, emerged [16]. Taking advantage of the development of the Italian Glycemic Index Table (unpublished data, Brighenti F, Department of Public Health, University of Parma, [19], [20]), we had the possibility to evaluate, in our original dataset, the role of GI and GL in relationship to MBD. We have also conducted a more detailed analysis of the possible influence that carbohydrates, overall and by subtype can have on MBD.

## Materials and Methods

### Ethics Statement

The study was approved by the local Florence Ethical Committee (Azienda U.S.L. 10 Firenze). All the study participants signed an informed consent and gave the permission to use the data collected during the study.

### Study Design

The design of the study has been already reported in detail [16]. Briefly, among the 10,083 adult women enrolled in the Florence section of the EPIC study between January 1993 and March 1998, we identified 2,001 participants with a mammographic examination (ME) negative for cancer diagnosis performed 5 (±1) years after enrolment and up to 31 December 1999. MEs had been originally carried out either in the frame of the local screening program or in a clinical setting.

### Mammographic Examination Retrieval and Breast Density Assessment

We retrieved most of the identified MEs directly from the Cancer Research and Prevention Institute (ISPO - formerly CSPO) archival files, but we also invited study participants to send us the ME films if these were kept at home. Overall, we were able to retrieve 1,668 (83.4%) MEs; the proportion of retrieval was higher for the films identified as stored in our archives (84.5%) than for those kept at home (75.6%). Women for which we were able to retrieve the original MEs, were older than women for which MEs were not obtained (53.4 years, SD 6.2 and 50.5, SD 6.4, respectively; *P*<0.001) and more frequently peri−/post-menopausal (70,5% and 56,1%, respectively; *P*<0,001). The oblique medio-lateral view of both breasts of all retrieved MEs were read independently by two experienced radiologists (DA ands MRdT) and MBD assessed according to Wolfe’s classification.

Disagreements between the two readers have been reviewed in joint sessions and a final classification has been reached and used for analyses [16].

### Data Collection

For all study women weight, height, waist and hip circumferences had been measured at enrolment (that is five years before the ME used for MBD assessment) by a trained nurse according to an international standard protocol.

Data on frequency of consumption of a large variety of food items and usual portion size had been obtained through a validated self-administered Food Frequency Questionnaire (FFQ) and then transformed into estimates of intake of a series of nutrients according to specifically developed Italian Food Tables [21], [22]. GIs of single food items containing available carbohydrates were obtained from the Italian Glycemic Index Table [unpublished data, Brighenti F, Department of Public Health, University of Parma] that lists over 150 food items covering 90% of the carbohydrate intake of people living in Italy. For food items not present in the table, GIs published elsewhere (International GI Tables [9] and www.glycemicindex.com) were used. A more detailed description of the entire procedure including the linkage with the EPIC-Italy FFQ has been published elsewhere [20].

The average dietary GI for each woman was calculated as the sum of the GIs of each food item consumed, multiplied by the average daily amount consumed and the percentage carbohydrate content, all divided by the total daily carbohydrate intake. The individual GL was calculated similarly except that there was no division by total carbohydrate intake. Each unit of GL represents a glycemic response equivalent to 1 g of glucose, with the total value representing the cumulative glycemic response in an “average” day.

A standardised lifestyle questionnaire collected detailed information on reproductive history, smoking and alcohol drinking history, exposure to environmental tobacco, medical history, educational level and other socio-economic and lifestyle variables. Information on drug use including use of Hormone Replacement Therapy (HRT), occupation and family history of cancer had also been collected [23].

In order to take into account exposure to a short term modifiers of MBD, updated information on use of HRT after enrolment were collected through a short mail questionnaire [16].

### Data Analysis

Women with high MBD (P2+DY according to Wolfe’s classification) were compared with women with low MBD (N1+P1), in line with other studies [15], [16], [24].

Estimates of GL, GI, total carbohydrate, simple sugar and starch intakes were adjusted for the energy intake of each woman using the regression-residual method [25] and classified into quintiles.

Crude and adjusted Odds ratios (OR) and 95% Confidence Intervals (CI) over energy-adjusted GI and GL quintiles were obtained. Multivariate logistic models were computed taking into account as potential confounders age (years), education (university and secondary school vs primary school), body mass index (overweight/obese), menopausal status (peri-menopausal women at enrolment were included in the postmenopausal group), number of children (0; 1–2; 3+), breast feeding (≤8 months; >8 months), non-alcohol energy intake (continuous), physical activity in leisure time (MET/week), fiber (quintiles), alcohol(quintiles) and saturated fat intake (quintiles). To calculate the p value for trend across quintiles, the median value of the specific quintile of consumption was considered and entered as a continuous term in the models. These multivariate analyses were carried out, after exclusion of women with missing values in the confounding variables, for 1,628 women.

Sensitivity analyses were performed after exclusion of women (n = 333) who reported to be dieting at the moment of enrolment, and women with a self-reported diagnosis of diabetes (n = 20) thus excluding a total of 353 subjects.

In order to evaluate the potential modifying effect of factors associated with hormone status and insulin resistance we performed analyses stratified for menopausal status at enrolment (yes/no) and BMI (<25 Kg/m^2^/≥25 Kg/m^2^). Tests of heterogeneity of the association were performed using indicator variables to model interaction terms between GI and GL (in quintiles) and BMI (normal weight vs overweight/obese) or menopausal status (yes/no). P-values were derived after comparison of the log-likelihood difference between models with and without interaction terms with a χ^2^-distribution with appropriate degrees of freedom.

We also evaluated the modifying effect of HRT use at the date of mammographic examinations (current HRT use yes/no) in the sub-group of women (1,589 in the multivariate analysis) for which this information was available.

The previously described analyses were also performed for overall carbohydrate intake and for intake of starches and simple sugars.

All analyses were performed using SAS statistical software (SAS/STAT version 9.2).

## Results

Overall, we were able to retrieve 1,668 MEs (83.4%) among those originally selected. Thus MBD was assessed for 1,668 women, with 1,103 (66.1%) classified as high (P2+DY) MBD and 565 (33.9%) as low (N1+P1) MBD, according to Wolfe’s classification.

Women in the high MBD category were younger, more educated, more frequently premenopausal and normal weight, in comparison with women classified in the low MBD category (Table 1). Moreover, women in the high MBD category reported a higher intake of total carbohydrates, starches and simple sugars and a higher GL in comparison with women in the low MBD category. The median GI was similar in the two categories. No relevant differences emerged in fiber and saturated fat intakes, while women in the high MBD category reported a higher alcohol intake (Table 2).

**Table 1:** Main characteristics of 1,668 participants by low and high MBD (EPIC-Florence).

	Mammographic Breast Density		
Main characteristics	Low	High	p-value**
**Age at enrolment (years)**			
<50	85 (19.5%)	351 (80.5%)	
50–59	379 (39.2%)	589 (60.8%)	
60+	101 (38.3%)	163 (61.7%)	<0.0001
**Education level**			
Primary school	213 (42.7%)	286 (57.3%)	
Secondary school	125 (38.1%)	203 (61.9%)	
High school	155 (26.8%)	423 (73.2%)	
University	72 (28.1%)	184 (71.9%)	<0.0001
**BMI Categories** *			
Underweight/normal (<25)	195 (22.3%)	680 (77.7%)	
Overweight (25–29.99)	254 (41.8%)	341 (58.2%)	
Obesity (≥30)	108 (62.4%)	65 (37.6%)	<0.0001
**Menopausal status** *			
Pre-menopausal	102 (20.8%)	389 (79.2%)	
Post-menopausal	463 (39.4%)	711 (60.6%)	<0.001
**Reproductive history** *			
Nulliparous	68 (27.6%)	187 (73.3%)	
Parous	497 (35.3%)	913 (64.8%)	0.26
**Number of children:**			
−1	134 (29.1%)	326 (70.9%)	
−2	255 (34.7%)	480 (65.3%)	
−3+	108 (50.2%)	107 (49.8%)	<0.0001
**Duration of breast feeding** *			
never	140 (32.9%)	285 (67.1%)	
≤8 months	204 (28.6%)	510 (71.4%)	
>8 months	220 (41.7%)	308 (58.3%)	<0.0001
Total	565 (33.9%)	1,103 (66.1%)	

* Due to missing data, some figures do not add up to the total.

** *P*-values from X^2^.

**Table 2:** Mean and median values of selected lifestyle and dietary variables of 1,668 participants by low and high MBD (EPIC-Florence).

	Mammographic Breast Density		
	Low	High	
Lifestyle and dietary variables	N = 565	N = 1,103	p-value**
**Leisure-time Physical Activity (MET hours/week)** *			
Mean	125.3	114.8	
Median (10°–90°)	126.0 (52.9–193.5)	110.2 (45.3–184.7)	<0.001
**Alcohol (g/day)**			
Mean	9.0	10.5	
Median (10°–90)	1.1 (0–24.2)	4.2 (0–26.0)	0.03
**Fiber (g/day)**			
Mean	21.3	21.9	
Median (10°–90°)	20.6 (13.3–31.1)	20.6 (13.5–31.3)	0.15
**Saturated fats**			
Mean	28.9	29.6	
Median (10°–90°)	27.0 (16.7–44.0)	27.8 (17.5–44.0)	0.24
**Non-alcohol energy intake (kcal/day)**			
Mean	2048.5	2127.4	
Median (10°–90°)	1993.8 (1315.9–2895.3)	2046.8 (1402.6–2952.3)	0.02
**Total carbohydrates (g/day)**			
Mean	254.0	269.9	
Median (10°–90)	243.9 (147.6–378.0)	255.0 (162.0–395.4)	0.001
**Starches (g/day)**			
Mean	154.3	165.1	
Median (10°–90°)	146.4 (70.8–239.6)	152.8 (84.4–258.5)	0.003
**Simple sugars (g/day)**			
Mean	99.5	104.5	
Median (10°–90°)	93.9 (56.1–152.7)	96.9 (57.6–161.0)	0.02
**Glycemic Index**			
Mean	54.9	55.2	
Median (10°–90°)	54.8 (51.2–58.4)	55.2 (51.7–58.6)	0.05
**Glycemic Load**			
Mean	139.5	147.6	
Median (10°–90°)	135.2 (80.4–203.8)	139.4 (86.9–218.8)	0.002

* Metabolic equivalents (METs) for non occupational physical activity (including recreational and household activities).

** *P*-values were calculated from GLM.

A positive association between GL and high MBD emerged in the comparison between highest and lowest quintiles in the crude model (OR = 1.50; 95%CI 1.07–2.11), and resulted strengthened in the adjusted model (OR = 1.73; 95%CI 1.13–2.67), with a borderline significant trend over quintiles (p = 0.048). No association emerged between GI and MBD (Table 3).

**Table 3:** Association between energy-adjusted GI, GL and high MBD (1,628 EPIC-Florence women).

			Crude model	Adjusted model
	Quintiles	Range	OR (95%CI)*	OR (95%CI)**
GlycemicIndex	I	<52.8	1	1
	II	52.9–54.3	0.92 (0.68–1.26)	0.91 (0.65–1.29)
	III	54.4–55.6	1.03 (0.75–1.40)	0.92 (0.65–1.30)
	IV	55.7–57.3	1.34 (0.97–1.84)	1.19 (0.83–1.70)
	V	>57.4	1.22 (0.89–1.68)	1.00 (0.70–1.43)
	p trend		0.06	0.63
GlycemicLoad	I	<101.8	1	1
	II	101.9–125.5	1.08 (0.78–1.49)	1.19 (0.83–1.69)
	III	125.6–151.1	0.87 (0.63–1.20)	0.98 (0.68–1.42)
	IV	151.2–185.0	0.89 (0.65–1.23)	1.02 (0.72–1.54)
	V	>185.1	1.50 (1.07–2.11)	1.73 (1.13–2.67)
	p trend		0.09	0.048

* Crude ORs obtained by models including only the variable of interest (classified in quintiles).

** Adjusted ORs obtained by multivariate logistic models including terms for age (years), education (university and secondary school yes/no), body mass index (normal weight/overweight/obese), menopausal status (pre−/post-menopausal), number of children (0; 1–2; ≥3), duration of breast feeding (≤8 months/>8 months), non-alcohol energy intake (kcal/day, continuous), leisure time physical activity (MET/week in continuous), alcohol (g/day), fiber (g/day) and saturated fat (g/day) intakes in quintiles.

When we performed the main analyses excluding women who reported at enrolment to be dieting or having been diagnosed with diabetes, a similar result was obtained: a significant association of increasing GL with high MBD was confirmed (highest versus lowest quintile OR 1.84; 95%CI 1.12–3.03, p for trend = 0.046).

Among the women for which information on HRT use at the date of ME utilized to assess MBD, was available, in a model further adjusted for current HRT use (yes/no), the highest quintile of GL was significantly associated with high MBD in comparison with the lowest quintile (OR 1.89; 95%CI 1.22–2.94) and a statistically significant trend emerged (p = 0.02). No association with GI was found.

The estimated crude and adjusted ORs for quintiles of energy-adjusted carbohydrates (overall and considering simple sugars and starches separately) and high MBD are reported in Table 4. A direct association between total dietary carbohydrates and high MBD emerged in the highest quintile of intake in comparison with the lowest quintile in the adjusted model (OR = 1.57; 95%CI 1.02–2.42) although the p for trend failed to reach the statistical significance (p = 0.07). A positive association emerged for simple sugar intake (OR = 1.71; 95%CI 1.13–2.59) in the comparison between highest and lowest quintile of intake with a significant trend over quintiles (p = 0.03). No significant association of increasing consumption of starches with MBD emerged. No association between carbohydrates, simple sugar and starch intake and high MBD emerged when the analyses were carried out excluding women who reported to be dieting or to have had a diagnosis of diabetes. When HRT use at the date of ME was included in the model a significant association also emerged with increasing intake of total carbohydrates (highest vs lowest quintile OR 1.71; 95%CI 1.10–2.66; p for trend = 0.03) and with intake of simple sugars (highest vs lowest quintile OR 1.80; 95%CI 1.17–2.80; p for trend = 0.02).

**Table 4:** Association between energy-adjusted total carbohydrate, simple sugars and starches and high MBD (1,628 EPIC-Florence women).

			Crude model	Adjusted model
	Quintiles	Range	OR (95%CI)*	OR (95%CI)**
Total carbohydrates (g/day)	I	<185.8	1	1
	II	185.9–228.4	1.11 (0.81–1.53)	1.21 (0.85–1.73)
	III	228.5–272.6	0.93 (0.68–1.27)	1.04 (0.72–1.49)
	IV	272.7–336.2	0.99 (0.72–1.36)	1.21 (0.82–1.79)
	V	>336.3	1.30 (0.94–1.80)	1.57 (1.02–2.42)
	p trend		0.26	0.07
starches (g/day)∧	I	<101.4	1	1
	II	101.5–133.5	1.03 (0.75–1.43)	1.06 (0.74–1.52)
	III	133.6–168.5	1.16 (0.83–1.61)	1.32 (0.90–1.93)
	IV	168.6–216.1	1.08 (0.78–1.51)	1.26 (0.85–1.89)
	V	>216.2	1.25 (0.88–1.79)	1.48 (0.93–2.35)
	p trend		0.22	0.08
simple sugars (g/day)∧	I	<69.4	1	1
	II	69.5–86.5	0.93 (0.67–1.30)	1.16 (0.81–1.66)
	III	86.6–104.2	0.92 (0.66–1.28)	1.22 (0.84–1.77)
	IV	104.3–130.2	0.80 (0.57–1.12)	0.98 (0.67–1.44)
	V	>130.4	1.25 (0.87–1.78)	1.71 (1.13–2.59)
	p trend		0.38	0.03

* Crude ORs obtained by models including only the variable of interest (classified in quintiles).

** Adjusted ORs obtained by multivariate logistic models including terms for age (years), education (university and secondary school yes/no), body mass index (normal weight/overweight/obese), menopausal status (pre−/post-menopausal), number of children (0; 1–2; ≥3), duration of breast feeding (≤8 months/>8 months), non-alcohol energy intake (kcal/day, continuous), leisure time physical activity (MET/week in continuous), alcohol (g/day), fiber (g/day) and saturated fat (g/day) intakes in quintiles.

∧ Included simultaneously in the crude and adjusted models.

We also evaluated the association between energy-adjusted Gl and GL at enrolment and high MBD in analyses stratified by menopausal status and BMI (Table 5). Although no significant interactions emerged, a positive association in post-menopausal women was evident in the highest in comparison with the lowest quintile of GL. No specific associations emerged in pre-menopausal women.

**Table 5:** Association between energy-adjusted GI and GL and high MBD by menopausal status and BMI (1,628 EPIC-Florence women).

Menopausal status		Pre-menopausal	Post-menopausal
		OR (95%CI)*	OR (95%CI)*
Glycemic Index	I	1	1
	II	0.98 (0.46–2.09)	0.90 (0.61–1.32)
	III	1.04 (0.49–2.21)	0.88 (0.60–1.31)
	IV	0.66 (0.31–1.39)	1.45 (0.96–2.19)
	V	1.92 (0.81–4.55)	0.85 (0.57–1.27)
	P trend	0.15	0.81
Glycemic Load	I	1	1
	II	0.95 (0.45–2.01)	1.32 (0.88–1.98)
	III	0.55 (0.26–1.19)	1.16 (0.76–1.77)
	IV	1.02 (0.44–2.35)	1.06 (0.69–1.64)
	V	1.55 (0.59–4.05)	1.83 (1.12–2.99)
	P trend	0.48	0.07
**BMI**		**BMI <25.0**	**BMI ≥25.0**
		**OR (95%CI)** *	**OR (95%CI)** *
Glycemic Index	I	1	1
	II	0.77 (0.46–1.29)	1.03 (0.66–1.63)
	III	0.95 (0.56–1.59)	0.85 (0.54–1.36)
	IV	1.12 (0.65–1.94)	1.17 (0.73–1.88)
	V	1.13 (0.67–1.93)	0.87 (0.54–1.41)
	P trend	0.35	0.74
Glycemic Load	I	1	1
	II	1.09 (0.64–1.85)	1.26 (0.79–2.01)
	III	1.06 (0.61–1.83)	0.96 (0.59–1.58)
	IV	1.30 (0.73–2.31)	0.90 (0.54–1.49)
	V	2.74 (1.40–5.38)	1.36 (0.77–2.40)
	P trend	0.005	0.67

* Adjusted ORs obtained by multivariate logistic models including terms for age (years), education (university and secondary school yes/no), number of children (0; 1–2; ≥3), duration of breast feeding (≤8 months/>8 months), non -alcohol energy intake (kcal/day, continuous), leisure time physical activity (MET/week in continuous), alcohol (g/day), fiber (g/day) and saturated fat (g/day) intakes in quintiles and, alternatively, body mass index (normal weight/overweight/obese) and menopausal status (pre-/post-menopausal).

When we considered separately normal weight (BMI <25.0) and overweight/obese women (BMI ≥25.0), we found a significant associations of high MBD with GL among lean women (BMI <25.0), with a p values for trend <0.005, although no significant interaction emerged (Table 5).

In the stratum of lean women, a significant association also emerged with increasing intake of total carbohydrates (highest vs lowest quintile OR 2.70; 95%CI 1.37–5.30; p for trend = 0.002) of starches (OR 2.61; 95%CI 1.27–5.35; p for trend 0.005) and simple sugars (highest vs lowest quintile OR 2.45; 95%CI 1.29–4.64; p for trend = 0.02). Again no formal significant interaction by BMI was found. No interaction emerged by menopausal status (see Table S1).

Overall, no formally significant interactions were found by HRT use at the date of mammogram (reported by 4.9% of women).

## Discussion

In this study we found a positive association between dietary glycemic load and high mammographic breast density, while no association emerged when average glycemic index was evaluated. Although no formal interaction emerged, this association was more evident among lean women.

To our knowledge no other study, so far, has evaluated the effect of individual estimates of dietary GI and GL on MBD. Our results support a positive association between dietary GL and high MBD while no effect emerged for GI. GI and GL indicate different characteristics of the overall pattern of carbohydrate intake. GI provides information on the carbohydrate quality of all foods in relation to the rate of glucose absorption and in this population showed a limited variation. On the other hand, GL is a measure of the glycemic effect of all servings of foods consumed in an average day (thus also including the information on the quantity of carbohydrates consumed) and can be considered a more sensitive indicator of the dietary insulin demand [6].

A positive association for highest quintile of carbohydrates intake (overall and for simple sugars separately) and high MBD was also found particularly among lean women. A few studies have evaluated the role of carbohydrate intake on MBD with inconsistent results. No association between high MBD and carbohydrate intake emerged in women with a family history of BC [26], [17]. In a study carried out among women enrolled in the EPIC Norfolk cohort, a high intake of carbohydrates was associated with Wolfe’s high risk mammographic patterns [15]. In a study carried out in Japanese women, high carbohydrate intake was inversely associated with high MBD assessed through a quantitative computer based method [14]. However we have to consider that nutrient intakes are likely to derive from different foods in this far Eastern population in comparison with Western populations. In our previous paper on dietary determinants of MBD in which a wide range of foods and nutrients were considered, we did not find a specific effect of carbohydrates in a simpler model [16]. Recently a specific Italian Glycemic Index Table (that lists over 150 food items covering 90% of the carbohydrate intake of people living in Italy) has been made available thus allowing analyses based on this additional classification of dietary carbohydrates [19], [20]. We have taken this opportunity to perform further analyses on this relevant topic in relation to MBD and, quite interestingly, the quintile-based analyses of GI and GL pointed out that high GL diets may be related to high MBD. The same pattern emerged also in the quintile-based re-analyses for overall carbohydrates and for simple sugar intakes.

There is a growing interest in the possible role of insulin resistance and hyperinsulinemia in the development of BC. MBD is widely recognized as a strong risk factor for BC and is affected by most of well-known or suspected BC risk factor including modifiable risk factors as diet and physical activity. MBD is considered to represent the fibro-glandular component of the breast [4]. High GL diets may contribute to increased levels of insulin, IGF-I and sex hormones (both estrogens and androgens) thus increasing proliferation of mammary epithelium. Some studies have reported a positive association between circulating levels of IGF-I and breast density in premenopausal women [27]–[31] and to a lesser extent in postmenopausal women [30], although other studies did not find such a relationship [32], [33].

Although in our analyses formal tests for interaction did not reach the level of statistical significance, the association between carbohydrates (overall and considering starches and simple sugars separately), GL and high MBD appeared more evident in lean women. While these results could be due to chance, it is notable that some studies on BC risk showed a positive association between glycemic load and BC risk only in lean women [19]. It is possible that, in overweight/obese women some of the metabolic effects of high GL are already present because of their excess adipose tissue, while in lean women a high GL diet might play an independent role in modulating BC risk.

The major strength of our study includes the longitudinal design, with a five-year interval between baseline assessment of dietary and lifestyle habits and MDB measurement, the detail in information collected including the information on HRT use at the date of mammographic examination utilized to assess MBD, allowing to take into account also aspects that could affect MBD in a short time. It is noteworthy that also in the analyses taking into account the possible effect of recent HRT use on MBD, we were able to confirm all these associations. We are aware that relevant changes in BMI could also influence the MBD assessment but we did not have the possibility to obtain a new measurement of BMI at time of the study mammogram with the same standardized procedures that we used at enrolment. Based on the results of a more recent follow-up of all cohort members, we can hypothesize that over 5 years women had increased their weight of less than 1 kg on average [34]. It seems unlikely that this relatively small increase may affect the results.

We used specific GI values determined for the most widely consumed Italian foods. The FFQ was also designed taking into account specific Italian dietary habits and to specifically assess food items typically consumed in our country, characterized by a high consumption of different dishes rich in carbohydrates.

The use of a FFQ for dietary assessment can lead to measurement errors and consequently to attenuated risk estimates. Moreover, estimates of GI and GL from FFQ might not accurately reflect the glycemic and insulinemic effect of specific recipes and mixed foods. Other factors like cooking procedures and chewing time can affect these aspects and are not easily captured by a FFQ. We have only one diet measurement and therefore we could not take into account possible changes in diet occurred before or after the enrolment. However, our main result, the association between GL and high MBD, was confirmed also in analyses excluding all subjects who reported a diagnosis of diabetes or dieting, a subgroup that possibly experienced recent changes in diet.

In conclusion we observed in a large group of Italian women characterized by a traditional dietary pattern based on a variety of carbohydrate-rich foods, an association between high glycemic load and high intake of total and rapidly absorbed carbohydrates and increased mammographic breast density. The results of this study warrant further investigation in order to better clarify the role of diet on breast cancer mediated by breast density, with a specific focus on carbohydrates.

## Supporting Information

Table S1 **Association between energy-adjusted carbohydrate (overall and considering simple sugar and starch intakes, separately) and high MBD by menopausal status and BMI (1,628 EPIC-Florence women).**
(DOC)
