# Supplementary material for: Glycemic Index, Glycemic Load and Mammographic Breast Density: The EPIC Florence Longitudinal Study
Source: PLoS One. 2013 Aug 7;8(8):e70943. doi: 10.1371/journal.pone.0070943 (PMC3737230; doi:10.1371/journal.pone.0070943)
Supplement: Table S1 — Association between energy-adjusted carbohydrate (overall and considering simple sugar and starch intakes, separately) and high MBD by menopausal status and BMI (1,628 EPIC-Florence women). (DOC) [file pone.0070943.s001.doc]

Table S1 Association between energy-adjusted carbohydrate (overall and considering simple sugar and starch intakes, separately) and high MBD by menopausal status and BMI (1,628 EPIC-Florence women).

| **Menopausal status** |  | **Pre-menopausal** | **Post-menopausal** |
| --- | --- | --- | --- |
|  |  | **OR (95%CI) ^*^** | **OR (95%CI) ^*^** |
| Total carbohydrates (g/day) | I | 1 | 1 |
|  | II | 0.72 (0.35-1.52) | 1.42 (0.94-2.13) |
|  | III | 0.68 (0.31-1.48) | 1.16 (0.76-1.77) |
|  | IV | 1.63 (0.66-4.01) | 1.14 (0.73-1.77) |
|  | V | 1.17 (0.45-3.05) | 1.67 (1.02-2.74) |
|  | P trend | 0.42 | 0.12 |
| starches (g/day)^ | I | 1 | 1 |
|  | II | 1.12 (0.50-2.50) | 1.06 (0.71-1.59) |
|  | III | 1.03 (0.46-2.30) | 1.44 (0.93-2.22) |
|  | IV | 1.14 (0.47-2.74) | 1.31 (0.83-2.07) |
|  | V | 1.17 (0.43-3.13) | 1.57 (0.92-2.68) |
|  | P trend | 0.75 | 0.08 |
| simple sugars (g/day)^ | I | 1 | 1 |
|  | II | 1.24 (0.60-2.57) | 1.14 (0.75-1.73) |
|  | III | 1.29 (0.57-2.88) | 1.18 (0.77-1.81) |
|  | IV | 0.95 (0.43-2.13) | 0.96 (0.62-1.50) |
|  | V | 2.39 (0.91-6.28) | 1.61 (1.01-2.59) |
|  | P trend | 0.17 | 0.09 |
| **BMI** |  | **BMI <25.0** | **BMI ≥25.0** |
|  |  | **OR (95%CI) ^*^** | **OR (95%CI) ^*^** |
| Total carbohydrates (g/day) | I | 1 | 1 |
|  | II | 1.02 (0.61-1.72) | 1.44 (0.89-2.32) |
|  | III | 1.22 (0.70-2.11) | 1.00 (0.61-1.64) |
|  | IV | 1.73 (0.95-3.12) | 1.00 (0.59-1.67) |
|  | V | 2.70 (1.37-5.30) | 1.17 (0.66-2.08) |
|  | P trend | 0.002 | 0.98 |
| starches (g/day)^ | I | 1 | 1 |
|  | II | 1.05 (0.63-1.75) | 1.10 (0.67-1.81) |
|  | III | 1.48 (0.83-2.64) | 1.24 (0.75-2.05) |
|  | IV | 1.62 (0.89-2.95) | 1.07 (0.63-1.83) |
|  | V | 2.61 (1.27-5.35) | 1.02 (0.56-1.89) |
|  | P trend | 0.005 | 0.97 |
| simple sugars (g/day)^ | I | 1 | 1 |
|  | II | 1.31 (0.75-2.26) | 1.14 (0.70-1.84) |
|  | III | 1.40 (0.80-2.44) | 1.14 (0.68-1.89) |
|  | IV | 1.02 (0.59-1.77) | 1.09 (0.64-1.87) |
|  | V | 2.45 (1.29-4.64) | 1.49 (0.85-2.61) |
|  | P trend | 0.02 | 0.19 |

^*^ Adjusted ORs obtained by multivariate logistic models including terms for age (years), education (university and secondary school yes/no), number of children (0; 1-2; ≥3), duration of breast feeding (≤ 8months/ >8 months), non- alcohol energy intake (kcal/day, continuous), leisure time physical activity (MET/week in continuous), alcohol (g/day), fiber (g/day) and saturated fat (g/day) intakes in quintiles and, alternatively, body mass index (normal weight/overweight/ obese) and menopausal status (pre-/post-menopausal).

^Included simultaneously in the models.
